# Investigation of Fresh Gastric Normal and Cancer Tissues Using Terahertz Time-Domain Spectroscopy

**DOI:** 10.3390/ma13010085

**Published:** 2019-12-23

**Authors:** Roman Grigorev, Anna Kuzikova, Petr Demchenko, Artem Senyuk, Anna Svechkova, Abdo Khamid, Alexander Zakharenko, Mikhail Khodzitskiy

**Affiliations:** 1 THz Biomedicine Laboratory, ITMO University, 3 b Kadetskaya Line, St. Petersburg 197101, Russia; anna.kuzikova@yandex.ru (A.K.); petr.s.demchenko@gmail.com (P.D.); khodzitskiy@yandex.ru (M.K.); 2 Pavlov First Saint Petersburg State Medical University, 6–8 L’va Tolstogo str., St. Petersburg 197022, Russia; artem.senuk@mail.ru (A.S.); Svechkova-95@mail.ru (A.S.); dr_nauras@hotmail.com (A.K.); 9516183@mail.ru (A.Z.)

**Keywords:** terahertz time-domain spectroscopy, cancer diagnosis, gastric cancer, optical diagnostics, tissue investigation, optical properties of tissues

## Abstract

In recent times, terahertz (THz) technologies have been actively applied in many biomedical research work, including gastric cancer diagnosis. In order to provide an effective removal of tumor during surgery, it is necessary to clearly distinguish it from different membranes of the stomach. In this work, we reported an investigation of various normal and cancer fresh gastric tissues using terahertz time-domain spectroscopy in the reflection mode. Refractive index and absorption coefficient of moderately differentiated and poorly differentiated gastric adenocarcinomas, as well as both serosa and mucosa were obtained in the frequency range from 0.2 to 1 THz. All cancer tissues were distinguishable from normal ones. The influence of the morphology of the investigated tissues on the obtained optical properties is discussed. The obtained results demonstrated a potential of THz time-domain spectroscopy to discriminate a tumor from normal serous and mucous gastric membranes. Thus, this method might be applied to gastric cancer diagnosis.

## 1. Introduction

Gastric cancer is one of the leading causes in terms of mortality and the number of diseases throughout the world. It is estimated that in 2018 more than 1,000,000 diseases of the stomach cancer were diagnosed, and the number of deaths reached 783,000 [1]. Gastric cancer can be removed by the endoscopic resection method or surgical operation. The first one is more painless and safe for the patient, however, it can only be used at the early stages of cancer and after timely endoscopic diagnosis [2]. For advanced cancer, it is necessary to excise the tumor using surgery. During surgery treatment it is important to discriminate between cancer regions and delineate the boundaries between tumor and normal tissue. Accurate intraoperative diagnosis of cancer improves the efficiency of cancer resection and can save more healthy tissues as well as increase the survival of patients after surgery.

In recent years, terahertz (THz) techniques have been actively studied and applied in many areas, such as communications, security, biomedical diagnosis, health monitoring, quality-control applications, etc. [3]. Biomedical applications of THz techniques are possible, because THz radiation is very sensitive to polar molecules, such as water, and it is absorbed by them. This feature allows the application of THz methods in the diagnosis of various neoplastic diseases because the water content in tumors is often higher than that in normal tissues. Moreover, chemicals and biological molecules can be identified by their characteristic resonant peaks because of the characteristic energies of molecules’ rotational and vibrational motions that are located in the THz frequency range [4]. Additionally, in contrast to X-rays, THz radiation has a low photon energy, that allows the performance of harmless diagnostics. Thus, THz spectroscopy and imaging methods have already been successfully used to detect tumors with different morphology and localization [5]. In recent years, there has been a growing interest in research on digestive system cancer, using THz diagnostic methods [6], which is related to the demand of clear recognition of boundaries between normal and cancer tissues during surgery or endoscopy treatment. It is expected that these methods are able to replace other gastric cancer diagnosis methods, such as conventional endoscopy, X-ray computer tomography, magnetic resonance imaging (MRI), optical coherence tomography (OCT), and positron emission tomography (PET). In [7], fresh normal and early gastric cancer tissues of mouse stomach were investigated through peak-to-peak THz imaging. Authors obtained a correlation between THz and pathologically mapped images of samples and found that optical density was increased in the tumors due to the high water content. Additionally, results of the work showed that THz imaging can detect gastric adenocarcinoma from normal mucous tissues but cannot detect signet ring cell carcinoma. In other studies, researchers obtained the difference between normal and cancer paraffin-embedded human gastric tissues, using THz time-domain spectroscopy [8,9]. Due to embedding in paraffin blocks, it is possible to except the influence of water to the optical properties of samples, and thus, the obtained results depend only on the morphology of investigated tissues. THz spectroscopy has also been applied to obtain the refractive index and absorption coefficient, and the permittivity of fresh excised gastric tissues consisted of cancer, normal, and pathologically changed areas [10]. It was shown that the optical density of cancer is higher than those of normal tissues and is close to water. In [11], authors applied a terahertz spectral unmixing method to detect gastric adenocarcinomas with a different grade of tumor from normal tissue. The potential of this method in identification of gastric cancer through an evaluation of the absorption coefficient of the samples, as well as its applicability and limitations were demonstrated. Additionally, different types of THz endoscopes were created, which made it possible to discriminate between normal and cancer tissues in vivo [12,13]. It is expected that further development of THz technologies will make it possible to create new effective and commercially inexpensive devices for the diagnostics of digestive systems, based on the THz methods [14]. Thus, all of these studies showed a large potential of THz methods in gastric cancer diagnosis.

In the works described above, the authors mainly investigated tumors from mucosa, but it is also important to discriminate cancer from other types of gastric tissues, because invasion of advanced gastric tumors often reaches different membranes of the stomach, such as the submucosa, the muscularis propria, the subserosa, and the serosa [15]. Widely accepted surgery methods such as laparoscopy require diagnostic procedures of different gastric membranes, including the external membrane that consists of serosa. Recognition of cancer cells that can pull away from a tumor in both mucous and serous gastric membranes is important, because these cells can decrease the efficiency of treatment and cause the disease to reappear in future. Due to the possibility of recognizing small objects such as cells [16,17,18] as well as high spatial resolution, in comparison to other methods [6], THz spectroscopy is a perspective method for an accurate and effective intraoperative diagnosis of gastric cancer.

In this work, we studied ex vivo fresh mucous, serous, and cancer tissues of the stomach using THz time-domain spectroscopy. The refractive indices and absorption coefficients in the frequency range of 0.2–1 THz are presented. Cancer tissues contained gastric, moderately and poorly differentiated adenocarcinomas, which reached the serous stomach membrane from the mucous one. Thereby, we have investigated tumors localized in both mucosa and serosa and compared it with the corresponding types of normal tissues.

## 2. Materials and Methods

### 2.1. Sample Preparation

**Figure 1:**
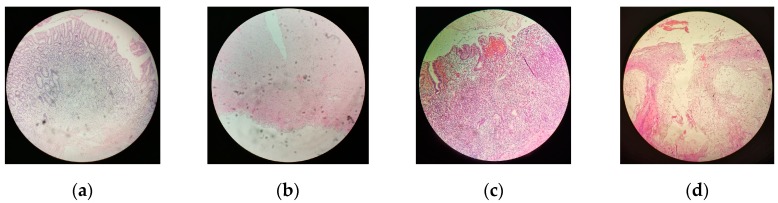
H & E-stained histopatolgy of gastric (**a**) mucosa; (**b**) serosa; (**c**) cancer localazed in mucosa; and (**d**) cancer invasion in serosa.

Normal and cancer fresh gastric tissues under this study were obtained from 4 patients with poorly and moderately gastric adenocarcinoma after gastrectomy, in the Pavlov First St. Petersburg State Medical University (St. Petersburg, Russia). All subjects gave their informed consent for inclusion before they participated in the study. The study was conducted in accordance with the Declaration of Helsinki, and the protocol was approved by the Ethics Committee of Pavlov First Saint Petersburg State Medical University (Project identification code is 224). Table 1 shows the information about patients and samples. Both normal and cancer tissues excised from patients passed a standard hematoxylin and eosin (H & E)-stained histology procedure (histopathology images are shown in Figure 1). Each sample of the normal tissue consisted of the mucosa on the one side and the serosa on the other side. Cancer tissues were excised from tumor localized in mucous and serous stomach membrane and had the same structure—the tumor tissue grown from the mucosa occupied one side, and a tumor that invaded into the serosa was on the other side.

After extraction, all samples were simultaneously placed into NaCl 9% physiological saline, in order to prevent dehydration of tissues. Until the start of the experiment, samples were in saline no more than 3 h. Solution residues were removed from the surface of the sample before measurements. The experiment was performed at a temperature of 17 °C, which is required for the experimental setup used in this work. The surface dimensions of the samples were at least 5 × 5 mm^2^.

### 2.2. Experimental Details

**Figure 2:**
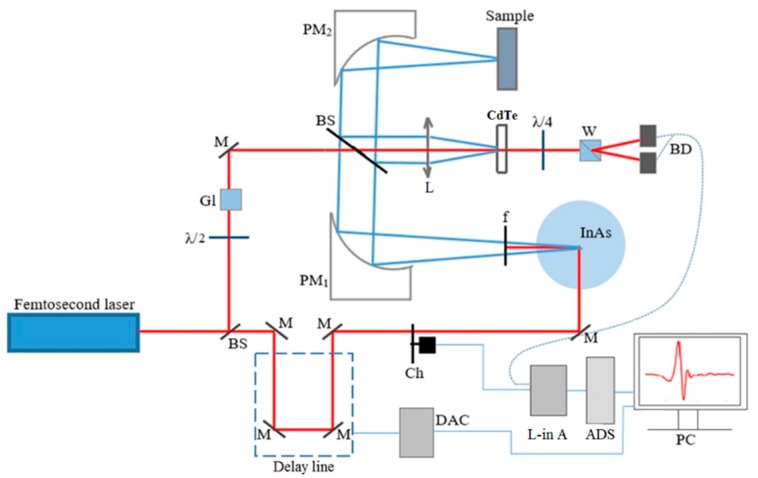
Schematic diagram of the THz TDS system in reflection mode. BS—beam splitter, M—mirror, Ch—chopper, PM—parabolic mirror, ADC—analog-to-digital converter, DAC—digital-to-analog converter, BD—balanced detector, L-in A—Lock-in Amplifier, W—Wollaston prism, f—filter, L—lens, λ/2—half-wave plate, and λ/4—quarter-wave plate.

The samples were investigated by THz time-domain spectrometer in the reflection mode. The working frequency range of the setup was from 0.2 to 1 THz and the maximal spectral resolution was 5 GHz. The scheme of the system is shown in Figure 2. InAs and CdTe crystals is used as a generator and a detector of THz radiation, respectively. Yb:KYW femtosecond laser provides pulses of 1,040 nm wavelength, 200 fs pulse duration, 75 MHz pulse repetition rate, and 1 W power. The pulses were divided into pump and probe beams by beam-splitter. The pump beam performed an optical excitation of InAs crystal to generate THz radiation with an average power of 30 µW and a power density of 60 µW/cm^2^. THz pulse was focused on a sample surface by a parabolic mirror and then reflected from the sample to the CdTe detector crystal. The THz and probe beams interacted at the detector. The probe beam obtained a linear polarization by a λ/2 plate and a Glan prism. Due to the Pockels effect, anisotropy was induced for the infrared beam, and it changed the polarization from linear to elliptical. A λ/4 plate changed the ellipticity ratio of the beams and Wollaston prism split the biased probe beam into s and p polarization components. A balanced detector was used to measure the difference of these components. The obtained signal was compared with the modulated signal by a lock-in amplifier, and when the modulation frequencies coincided, the final signal was amplified and registered. Each sample was examined at one point with a number of measurements at point 500 and number of registered waveforms at point 3, which were then averaged. Three waveforms were registered in approximately 15 min. Signal/noise ratio of the setup was 40 dB.

**Figure 3:**
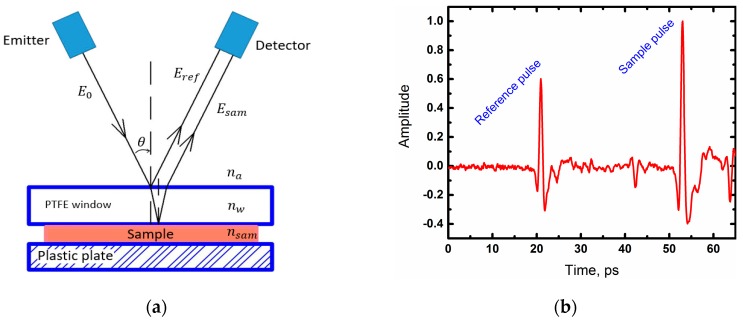
(**a**) Incident wave propagation in the double-reflection measurement method. ${\hat{E}}_{0}$—incident THz field, ${\hat{E}}_{ref}$, and ${\hat{E}}_{sam}$—the THz field reflected from the air—window and window-sample interface, respectively. $n_{a}$, $n_{w}$, and $n_{sam}$ are the refractive indices of air, window and sample, respectively; (**b**) THz time-domain waveform corresponding to a tissue (mucosa).

In order to measure the sample in reflection mode, a double-reflection measurement, also known as self-referenced geometry [19] was used (Figure 3a). In this method, the sample should be placed under the special dielectric window. Incident THz beam reflected from the top surface of the window (‘air-window’ interface) and reached the detector. Another part of the beam penetrated into the window, refracted, reached the sample located under the window (‘window-sample’ interface) and reflected from it. The intensity and phases of the beam reflected depended on the optical properties of the sample. A waveform of both reference and sample pulses is shown in Figure 3b.

**Figure 4:**
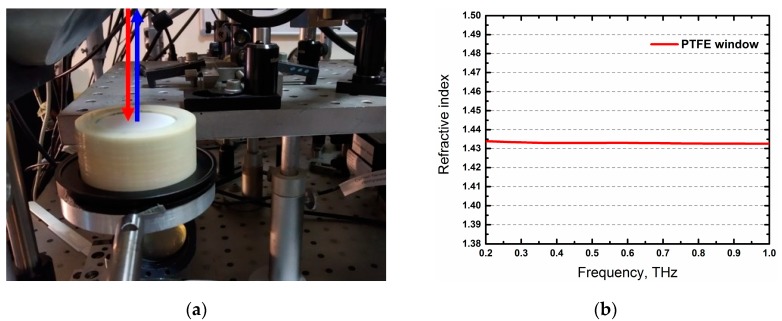
(**a**) The sample holder with the polytetrafluoroethylene (PTFE) plate used as a window and (**b**) refractive index of PTFE.

To obtain a high reflection coefficient from ‘window-sample’ interface, the refractive indices of the window and the sample must be different and as strong as possible. Since typical refractive index of fresh biological tissues is approximately between 2.8 and 2.0 in the frequency range of 0.2–1 THz, the refractive index of the window should be higher or lower than these values, whenever possible. Additionally, this material should not absorb a high amount of THz radiation. Thus, polytetrafluoroethylene (PTFE), also known as Teflon, with a thickness of 3360 μm was chosen to fabricate a window for the experiment. The window was set in a sample holder shown in Figure 4a. The incident angle of the pulse was normal to the surface of the window, which provided for an easy and accurate data analysis discussed in the next section. PTFE has a low absorption in the THz frequency range, and its refractive index is about 1435 (Figure 4b), which is less than the refractive index of most fresh biological tissues. All of this allows the obtainment of a good reflection of the signal from the “window–sample” interface and, therefore, allows the detection of THz signals of high amplitudes.

### 2.3. Data Analysis

As mentioned above, the reflected THz signal is composed of two pulses—the first pulse is from the air/window interface that is named as the ‘reference pulse’, and the second one is a delayed pulse reflected from the window/sample interface that is named as ‘sample pulse’. In order to obtain the optical properties of the samples, it is necessary to transform the waveforms into the frequency domain using Fast Fourier Transform (FFT). Since the waveform includes both reference and sample signals, it is necessary to use window functions to distinguish each of them. As the differences between different types of window functions are negligible in frequencies below 1 THz, a Gaussian window was chosen. This window provided a moderate improvement for both spectral and dynamic ranges [20]. As a result, complex amplitudes for the reference signal ${\hat{E}}_{ref}$ and the sample signal ${\hat{E}}_{sam}$ were obtained.

The transfer function was calculated by dividing the complex amplitude of the sample signal by the complex amplitude of the reference signal [19]:

(1) $$\hat{H}=\frac{{\hat{E}}_{sam}}{{\hat{E}}_{ref}}=\frac{\tau_{aw}\tau_{wa}}{\rho_{aw}}\exp\left[-2in_{w}\frac{wl}{c}\right]\frac{n_{w}-{\hat{n}}_{sam}}{n_{w}+{\hat{n}}_{sam}},$$

where ${\hat{E}}_{ref}$ and ${\hat{E}}_{sam}$ are the complex amplitudes of the reference and the sample signals, respectively, *l* is the window thickness, $\tau_{aw}$ and $\tau_{wa}$ are the transmission coefficients for the air–window and window–air interfaces, respectively, $\rho_{aw}$ is the reflection coefficient of the air–window interface, $\rho_{aw}$ is the reflection coefficient of the air–window interface, $n_{w}$ is the refractive index of a window, ${\hat{n}}_{sam}=n_{sam}-jk_{sam}$ is the complex refractive index of the sample, and *c* is the speed of light. The refractive index of a window was found using the formulas described in [21].

The transmission and the reflection coefficients for normal angle of incidence are given by

(2) $$\tau_{aw}=\frac{2}{1+n_{w}},$$

(3) $$\tau_{wa}=\frac{2n_{w}}{1+n_{w}},$$

(4) $$\rho_{aw}=\frac{1-n_{w}}{1+n_{w}}.$$

Rearranging (1) gives [19]

(5) $$\frac{n_{w}-{\hat{n}}_{sam}}{n_{w}+{\hat{n}}_{sam}}=\frac{\rho_{aw}}{\tau_{aw}+\tau_{wa}}\exp\left[2in_{w}\frac{wl}{c}\right]\hat{H}=Aexp\left(i\varphi \right),$$

where A and φ are the amplitude and phases of the complex Fresnel coefficient from the window/sample interface, respectively.

Finally, solving Equation (5) can help determine the refractive index and extinction coefficient of sample [21]:

(6) $$n_{sam}=\frac{n_{w}\left(1-A^{2}\right)}{1+A^{2}+2Acos\varphi },$$

(7) $$k_{sam}=\frac{2n_{w}Asin\varphi }{1+A^{2}+2Acos\varphi }.$$

It is possible to calculate the absorption coefficient using the extinction coefficient:

(8) $$\alpha \left(\omega \right)=2\frac{\omega k_{sam}}{c}.$$

## 3. Results and Discussion

**Figure 5:**
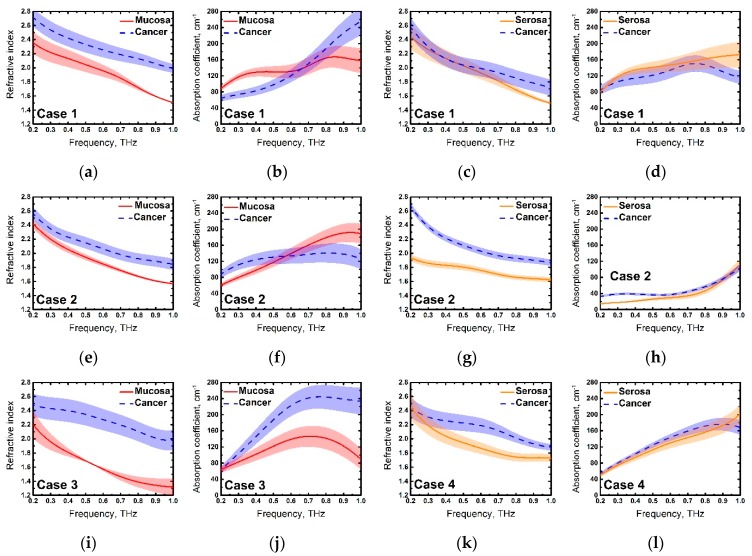
Refractive index and absorption coefficient of mucous, serous gastric fresh tissues, and cancer localized in both mucous and serous membranes of the stomach: (**a**–**d**) Case 1; (**e**–**h**) Case 2; (**I**,**j**) Case 3; and (**k**,**l**) Case 4.

Obtained optical properties of the samples are shown in Figure 5.

In general, cancer could be well distinguished from both mucous and serous membranes, especially, by the refractive index. In some cases, the errors of absorption were very high (Figure 5d,h,l), which did not allow to accurately diagnose the tumor. Instability in absorption coefficient could also be observed in Figure 5b,f where the curves intersected after 0.5 THz. Basically, cancer and normal tissues could be well-distinguished by absorption coefficient only in Case 3 (Figure 5j). The strong deviation of absorption coefficient sometimes occurred when investigating fresh biological tissues by the THz time-domain spectroscopy, which was also obtained by other authors [22,23,24]. On the other hand, the difference between cancer and normal tissues was clearly seen by the refractive index in all cases, except Case 1 (Figure 5c), where cancer and serosa were distinguishable only above 0.6 THz. It is worth noting that the most noticeable difference in the optical properties of healthy and tumor tissues in all cases (except Figure 5h,l) was also observed above 0.6 THz. We suggest that the reason for this might be due to the strong water-absorption lines lying above this frequency [25].

**Figure 6:**
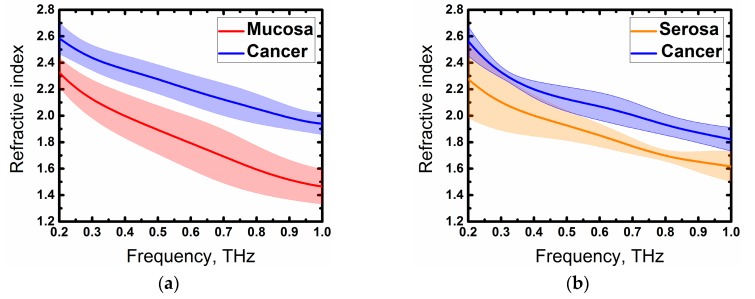
Refractive index of the investigated tissues from (**a**) mucous membrane and (**b**) serous membrane averaged of all cases.

Thus, we have obtained the expected results—optical density and absorption of cancer tissue, in general, were higher than that of both mucosa and serosa, which corresponded to other research on cancer, using THz time-domain spectroscopy [8,9,10,11]. This difference could be explained by the high cell density in cancer regions [5], features of the tumor microenvironment that cause profound metabolic changes in the cells [26,27], abnormal protein density alterations, and the increase in the vasculature [27]. However, one of the main factors affecting the THz response from fresh biological tissue is an increased water content in tumors in comparison to normal tissues [8,22]. As seen in Figure 3, values and relations of dispersions of refractive indices and absorption coefficients might vary slightly in different cases, this could be explained by the changing water concentration in the samples that depend on the individual particularity of different patients as well as other factors, such as the difference in time during which the samples were in the NaCl saline. In spite of this, the factors described above, especially increased the water content in cancer cells, making a more significant contribution to a response of the THz pulse from tissues. In order to demonstrate this, we averaged the values of the refractive indices of four measurements with one standard deviation and showed the resulting dispersions in Figure 6. Normal and cancer tissues were distinguishable from both mucous and serous gastric membranes. Notably, cancer tissues from both membranes had roughly the same optical density, which corresponded to the fact that the morphology of the tumor did not depend on the invasion of different gastric membranes. Additionally, we were unable to differentiate between moderately differentiated and poorly differentiated adenocarcinomas. Since increased water content is a symptom of many diseases, it is possible to confuse them with cancer during THz diagnosis. In [22], the authors did not find the difference in gelatin-embedded human brain gliomas with different World Health Organization (WHO) grades and edematous tissues in the brain. Additionally, the morphology of squamous cell carcinomas (SCC) and basal cell carcinomas (BCC) was not distinguished by THz imaging, unlike optical imaging that accurately presents the morphological features [28]. Authors concluded that THz diagnosis methods might be combined with other methods to obtain information about both the morphology and location of the tumor. Thus, it is possible to detect a disease using THz spectroscopy or imaging, but identification of its types can be hampered.

As the penetration depth of THz beam into biological tissues is limited, we were able to obtain information only about the top layers [3]. Using the formula $\delta \left(\omega \right)=\frac{1}{\alpha \left(\omega \right)}$, we averaged the absorption coefficients of all measurements (shown in Figure 5), separately for mucosa and serosa, and obtained the tissue penetration depths that were approximately 65 ± 15 µm at 0.9 THz for mucosa and 84 ± 40 µm at 0.9 THz for serosa. The structure and morphology of the top mucous layer were different from that of the serosa, therefore, the influence of the interacted THz waves was also different. The surface of the mucosa consisted of foveolar cells (surface mucous cells), which are constituted of a simple columnar epithelium. These cells contain water and proteoglycan as well as produce mucus [29]. The serous membrane comprises connective tissue covered by a secretory epithelial layer, which secretes lubricating and transport fluids [30]. Since there are epithelium layers in both serosa and mucosa, we have obtained similar optical properties. Unfortunately, the strong deviation, which could have been due to the different hydration of the tissues, as well as the secreted fluids mentioned above, did not allow a comparison of the optical properties of mucosa and serosa, with a high accuracy. Nevertheless, cancer is well distinguishable from, both, mucosa and serosa, which is the main requirement in an intraoperative diagnosis of malignant neoplasms. In future works we are planning to investigate THz response of various types of fresh gastric tissues, specifically, by conducting more statistical analyses, reducing the tissue dehydration by gelatin embedding [31], as well as using theories that introduce an inhomogeneous nature of biological tissues [22].

The obtained results demonstrated the possibility of diagnosing moderately and poorly differentiated gastric adenocarcinomas in both the serous and mucous membranes of the stomach. Further studies should be concentrated on the detection of small cancer areas in different gastric tissues, using THz spectroscopy and imaging. The final step is a development of portable and inexpensive THz device with a high detection efficiency to perform gastric cancer diagnosis in a clinical setting.

## 4. Conclusions

In this work, we investigated different types of fresh gastric tissues, with and without tumor invasions, using THz time-domain spectroscopy. The refractive indices and absorption coefficients of serosa, mucosa, moderately differentiated and poorly differentiated gastric adenocarcinomas were obtained. We demonstrated that cancer tissues showed higher optical properties than that of normal tissues, and in general they could be distinguished from both the mucous and serous membranes of the stomach. Additionally, tumor is best distinguishable from both mucosa and serosa through the refractive index. The presented results indicated that THz spectroscopy could be a potential tool for non-invasive effective intraoperative diagnosis of gastric cancer. Further investigations with clinical studies and large statistics allowed a deeper understanding of the possibilities of the THz methods.

## Figures and Tables

**Table 1:** Information about patient and sample characteristics.

Case	Age	Gender	Tissues	Pathology (TNM)
1	71	Female	Mucosa and serosa	pT4aN2M0G3
2	74	Female	Mucosa and serosa	pT3N0M0G2
3	74	Male	Mucosa only	pT2N0M0G2
4	83	Female	Serosa only	pT3N3bM0G3

pT3N0M0G2 and pT2N0M0G2 are moderately differentiated gastric adenocarcinomas, Grade 2; pT4aN2M0G3 and pT3N3bM0G3 are poorly differentiated gastric adenocarcinomas, Grade 3.
